# Trends of lip, oral cavity and oropharyngeal cancers in Australia 1982–2008: overall good news but with rising rates in the oropharynx

**DOI:** 10.1186/1471-2407-13-333

**Published:** 2013-07-06

**Authors:** Anura Ariyawardana, Newell W Johnson

**Affiliations:** 1 Population and Social Health Research Programme (Population Oral Health Group), Griffith Health Institute, Gold Coast Campus, Griffith University, Building G05, Room 3.22A, Gold Coast, QLD 4222, Australia; 2 School of Medicine and Dentistry, James Cook University, Building D1, Cairns Campus, Smithfield, QLD 4870, Australia

**Keywords:** Lip cancer, Oral cancer, Oropharyngeal cancer, Epidemiology, Trends, Australia

## Abstract

**Background:**

Considerable global variation in the incidence of lip, of oral cavity and of pharyngeal cancers exists. Whilst this reflects regional or population differences in risk, interpretation is uncertain due to heterogeneity of definitions of sites and of sub-sites within this anatomically diverse region. For Australia, limited data on sub-sites have been published. This study examines age-standardised incidence trends and demography from 1982 to 2008, the latest data available.

**Methods:**

Numbers of cases within ICD10:C00-C14 were obtained from the Australian Institute of Health and Welfare, recorded by sex, age, and sub-site. Raw data were re-analysed to calculate crude, age-specific and age-standardised incidence using Segi’s world-standard population. Time-trends were analysed using Joinpoint regression.

**Results:**

Lip, Oral Cavity and Pharyngeal (excluding nasopharynx) cancers, considered together, show a biphasic trend: in men rising 0.9% pa from 1982 to 1992, and declining 1.6% pa between 1992 and 2008. For females: rises of 2.0% pa 1982–1997; declines of 2.8% pa 1997–2008. Lip cancer is declining especially significantly. When the Oropharynx is considered separately, steadily increasing trends of 1.2% pa for men and 0.8% pa for women were observed from 1982 to 2008.

**Conclusions:**

Although overall rates of lip/oral/oropharyngeal cancer are declining in Australia, these are still high. This study revealed steady increases in cancers of the oropharynx, beginning in the late 1990s. Continued efforts to reduce the burden of these cancers are needed, focused on reduction of the traditional risk factors of alcohol and tobacco, and with special emphasis on the possible role of human papillomavirus and sexual hygiene for cancers of the oropharynx.

## Background

Cancer is a growing public health problem worldwide. Overall, 12.4 million new cancer cases and 7.6 million deaths were reported to have occurred in 2008 [1]. Of these, estimates of 263,000 new cases of lip and oral cavity cancers, and 135,000 cases of pharyngeal cancers (excluding nasopharynx) were reported, representing 2.1% and 1.1% of all new cancers respectively [2]. A large majority of cancers of the upper aero-digestive tract, excluding the nasopharynx, are squamous cell carcinomas. Cancers of the lip, tongue and oral cavity (ICD-10:C00-C06) and of the oropharynx (ICD-10:C09, C10 and C14) have several risk factors in common, have similar biology and are often grouped together [3]. A 20-fold global variation in the incidence of these cancers is apparent in international databases [2,4]. Two-thirds of the burden is within the developing world, where under-ascertainment of cases is significant [5]. Importantly, some of the highest rates are seen in parts of Western and Eastern Europe and the former Soviet republics [4].

The considerable variation in the pattern of oral and of oropharyngeal cancer incidence in different parts of the world reflects differences in the prevalence of specific risk factors. A high incidence of lip cancer is found among white races exposed to solar radiation. High rates of incidence of cancers of intra-oral sites are reported from communities with high consumption of tobacco, particularly among users of smokeless tobacco, often in association with areca nut in the form of betel quid: here, carcinogenesis may also be synergised by high consumption of alcohol [6-8]. A rising incidence of lip, of oral cavity and of pharyngeal cancers, taken together (ICD 10: C00-C14), has been reported in some industrialised countries since the 1970s: Statistically significant increases of 18% and 30% were observed from 1990 to 1999 in the UK for males and females respectively [9]. A recent study in Denmark reported an overall rise in head and neck cancer incidence between 1978 and 2007, particularly for the oral cavity (2.2% pa), tonsil (4.8% pa), and oropharynx (3.5% pa) [10]. A significant increase in the incidence of cancer of the oropharynx (C01, C05.1, C05.2, C09, C10, C12. C13 and C32) was observed during the period from 1989 to 2006 in the Netherlands, at the rate of 2.5% and 3.0% per year in males and females respectively [11]. In contrast to this, declines in lip plus oral cavity plus pharyngeal cancer mortality rates have been reported in several countries e.g. USA, China, Hong Kong, Italy, Spain, France, Germany and Australia [12]: with these grouped data, much of the effect is due to reduction in cancer of the nasopharynx which is, biologically, a distinctly different disease than that of most of the upper aerodigestive tract. In addition to the traditional risk factors, recent data from some western countries suggest that humanpapillomaviruses (HPV) are responsible for a rising incidence of oropharyngeal cancers [13-15]. A recent study in Australia has also shown increasing trends in potentially HPV-associated cancers of the oropharynx [16].

Literature on the incidence of oral and of oropharyngeal cancer in Australia is scarce, especially relating to sub-sites within ICD10:C00-C14. In 1971, Tan reported the countrywide incidence of lip cancer for the period 1959 to 1964. This hospital-based study found a decline of lip cancer incidence (upper and lower lip combined) from 6.5/100,000 in 1959 to 4.9/100,000 in 1964 [17]. Macfarlane et al., in 1994, reported patterns of oral and pharyngeal cancer incidence in New South Wales based on the population-based cancer registry in that jurisdiction. They found increasing trends of “oral and pharyngeal cancer” from 6.5/100,000 pa for males and 2.1/100,000 pa for females, respectively, in 1974 to 9.3/100,000 pa for males and 3.0/100,000 pa for females in 1986. It appears, however, that this trend has not continued thereafter [18].

A report from the population-based South Australian Cancer Registry revealed marginally increasing trends of tongue cancer in males from 0.98/100,000 pa between 1977 and 1985, rising to 1.15/100,000 pa between 1994 and 2001: the incidence in females was, however, stable for the same period at 0.45/100,000 pa [19]. Abreu et al., in 2009, described an upward trend in the incidence of lip cancer in Western Australia with rates of 8.9/100,000 pa and 2.7/100,000 pa for males and females respectively, although these data are based on a small population [20]. Another study in Western Australia reported increasing trends in “oral and pharyngeal” cancer between 1982 and 1990 peaking at 14.6/100,000 pa for males and 6.2/100,000 pa for females, with declining trends thereafter [21].

Interpretation of the available literature is uncertain, due to heterogeneity of definitions of lip, oral cavity and of oropharyngeal cancer. To the best of our knowledge, no literature is available on recent trends of lip, of oral cavity and of pharyngeal cancers across Australia, based on strict sub-site analyses. The aim of the present paper is, therefore, to describe age-standardised incidence, trends and demography of sub-sites of lip, of oral cavity and of oropharyngeal cancers (ICD10:C00-C14, excluding C11, the nasopharynx) from 1982 up to the most recent data available, namely 2008.

## Methods

The numbers of cases of head and neck cancers were obtained for the period 1982 to 2008 from the Australian Institute of Health and Welfare (AIHW). The AIHW compiles the Australian Cancer Database, a collation of all primary malignant neoplasms diagnosed in Australia. This is compiled from data provided by state and territory cancer registries through the Australian Association of Cancer Registries. Population-based cancer registries receive information on cancer diagnoses from a variety of sources: hospitals; pathology laboratories; radiotherapy centres; and registries of births, deaths and marriages.

The data were segregated by sex, age, and anatomical site based on the World Health Organisation International Classification of Diseases for Oncology, 3rd edition (ICD-O-3) ICD-10 codes. Age was grouped into 5-year bands 0–4, 5–9, 10–14, 15–19, 20–24, 25–29, 30–34, 35-39, 40–44, 45–49, 50–54, 55–59, 60–64, 65–69, 70–74, 75–79, 80–84 and 85+. Annual mid-year population estimates for the period by age group and sex were obtained from the Australian Bureau of Statistics [22].

Cancers in the present analysis are: “Lip and Oral cavity”, which includes lip (ICD 10; C00); the Oral Tongue (Anterior two-thirds only; C02); Gum (C03); Floor of mouth (C04); Hard Palate (C05.0) and other unspecified parts of mouth (C06). Cancers of the Base of the tongue (C01), Soft palate (C05.1), Uvula (C05.2), Tonsil (C09), Oropharynx (C10) and other ill defined sites of oral cavity and pharynx (C14) were considered separately as cancers of the “Oropharynx”. Malignant neoplasms of salivary glands (C07, C08) and other pharyngeal sites (Naso- and Hypo-pharynx: C11-13) were excluded.

Raw data were re-analysed to calculate crude, age-specific and age-standardised incidence rates. Segi’s world standard population and the direct method were used to calculate age-standardised incidence rates [23].

Time trends in age-standardised incidence rates were analysed using Joinpoint regression modeling [24]. The Joinpoint programme version 3.5.2 was used [25]. This analysis generates discrete points that separate different line segments on a log scale, to describe the trends over time. The analysis involves 0–4 “Joinpoints” and the Monte Carlo permutation method to test the level of significance of the trends. Annual percentage change (APC) of each segment, and annual average of APC with corresponding 95% confidence intervals, were estimated. APC was tested to determine whether the trends are increasing (positive change) or decreasing (negative change). P values of <0.05 were considered statistically significant.

## Results

A total of 56,226 cases (40,163 in men and 16,063 in women) of lip, of oral cavity and of oropharyngeal cancers were reported during the period from 1982 to 2008. Table 1 shows the age standardised incidence (ASI) rates by major sites of the cancers for the beginning (1982) and the end (2008) of the study period and the results of the Joinpoint regression analysis for males, females and both sexes together.

**Table 1:** Lips, Oral cavity and oropharyngeal cancer incidence rates for Australian for major sites from 1982–2008 by sex

**Site**	**Sex**	**Year 1982**	**Year 2008**	**Joinpoint analysis (1982–2008)**				
				**Trend 1**		**Trend 2**		**AAPC**
		**ASI (n)**	**ASI (n)**	**Period**	**APC**	**Period**		**1999-2008**
Lip Oral cavity and Oropharynx ICD10 C00-06,C09,C10, and C14	Male	13.67 (1139)	11.01 (1709)	1982-1992	0.9	1992-2008	−1.6*	−1.6*
	Female	4.06 (343)	4.07 (756)	1982-1997	2.0*	1997-2008	−2.8*	−2.8*
	Both sexes	8.34 (1482)	7.47 (2465)	1982-1992	1.6*	1992-2008	−1.4*	−1.4*
Lip and Oral cavity ICD10, C00-C06, (Excluding C01)	Male	10.84 (904)	7.04 (1107)	1982-1994	0.5	1994-2008	−3.1*	−3.1*
	Female	3.27 (277)	3.06 (584)	1982-1996	2.3*	1996-2008	−3.0*	−3.0*
	Both sexes	6.62 (1181)	5.01 (1691)	1982-1995	0.9*	1995-2008	−2.8*	−2.8*
“Oropharynx” ICD10; C01, C05.1, C05.2, C09.07, C091.1, C09.8, C09.9, C10 and C14	Male	2.83 (235)	3.97 (602)	1982-2008	1.2*			1.2*
	Female	0.79 (66)	1.01 (172)	1982-2008	0.8*			0.8*
	Both sexes	1.72 (301)	2.46 (774)	1982-2008	1.2*			1.2*

Abbreviations

*APC*, Annual Percent Change, *AAPC*, Average Annual Percent Change.

*The Annual Percent Change (APC) is significantly different from zero at alpha = 0.05.

*The Average Annual Percent Change (AAPC) is significantly different from zero at alpha = 0.05.

Overall, ASI for Lip, Oral Cavity and Oropharyngeal cancers combined (C00-06, C09, C10 and C14) for males declined from 13.67/100,000 in 1982 to 11.01/100,000 in 2008. Female rates were lower, but stable: 4.06/100,000 in 1982 and 4.07/100,000 in 2008. When both sexes are considered together, the ASI for 1982 was 8.34/100,000 and 7.47/100,000 for 2008.

**Figure 1:**
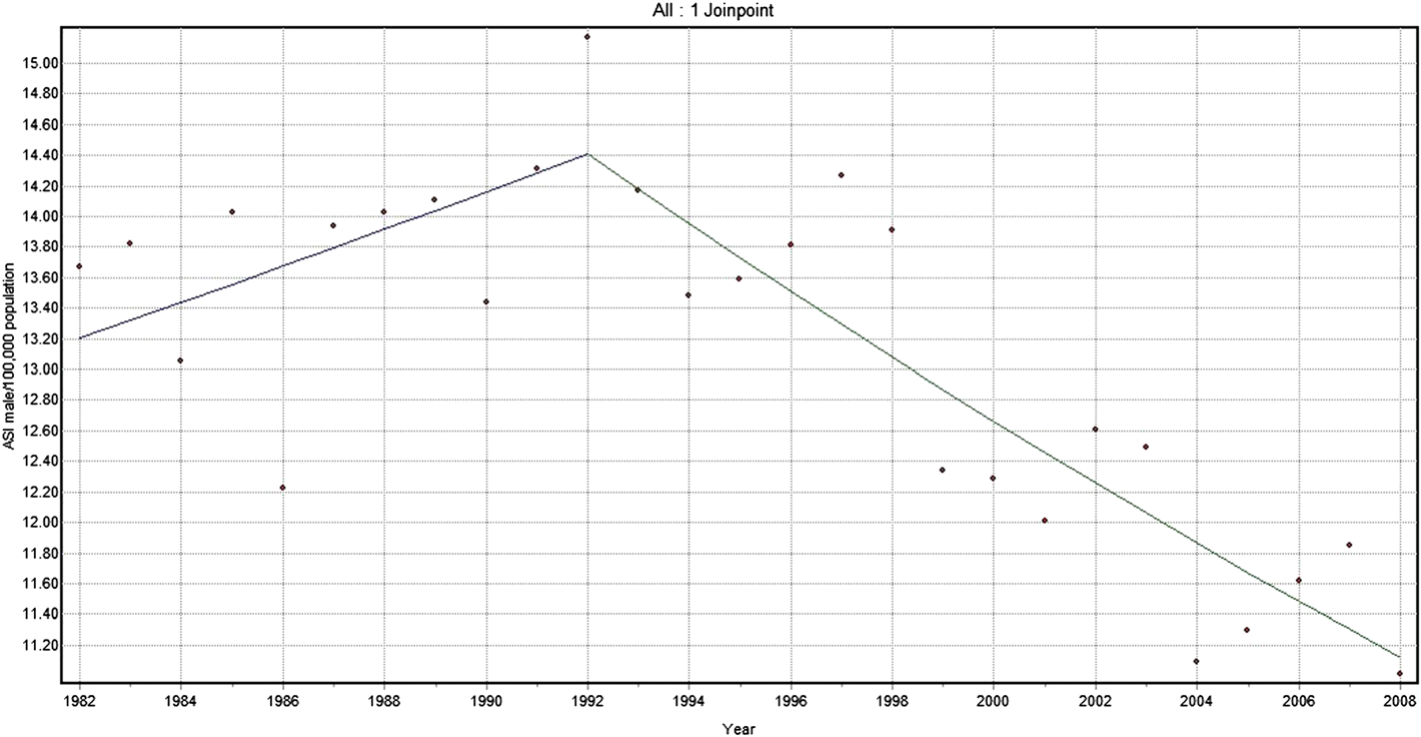
Trends in the incidence of lip, oral cavity and oropharyngeal cancer (C00-06, C09, C10 and C14), Male 1982–2008.

**Figure 2:**
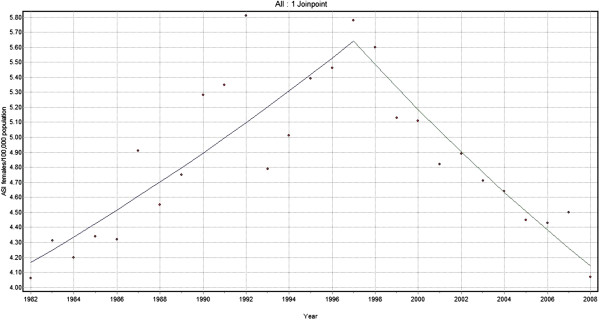
Trends in the incidence of lip, oral cavity and oropharyngeal cancer (C00-06, C09, C10 and C14), Female 1982–2008.

Joinpoint analysis for Lip, Oral Cavity and Oropharyngeal cancers combined for men showed an increasing trend of 0.9% per year from 1982 to 1992, followed by a decline of 1.6% per year from 1992 to 2008 (Figure 1). Similar biphasic trends were demonstrated for females, from a lower base, and with a 5 year time lag: a steady increase in trend of 2.0% per year from 1982–1997 followed by a sharp decline of 2.8% per year from 1997–2008 (Figure 2). Consideration of both sexes together for the same cancers reveals an increasing trend with annual change of 1.6% from 1982 to 1992 and a declining trend with 1.4% annual change from 1992 to 2008.

When Lip plus Oral cavity cancers (C00-06) are considered separately from the pharynx there was a decline for males from 10.84/100,000 pa in 1982 to 7.04/100,000 in 2008: for females from 3.27/100,000 pa in 1982 to 3.06/100,000 in 2008 and for both sexes from 6.62/100,000 to 5.01/100,000 in 2008 (Table 1).

**Figure 3:**
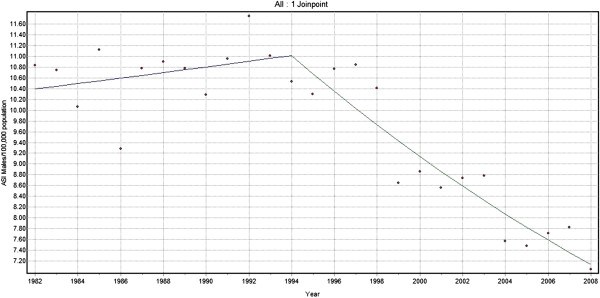
Trends in the incidence of lip and oral cavity cancer (C00-C06) excluding base of the tongue (C01), Males 1982–2008.

**Figure 4:**
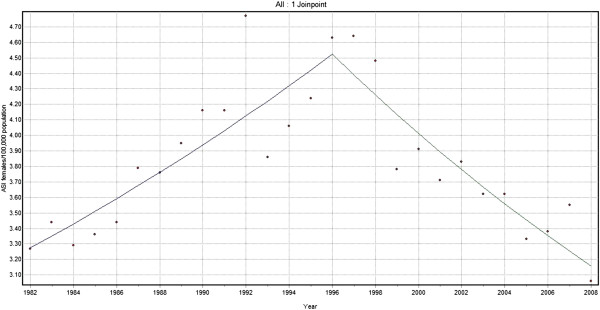
Trends in the incidence of lip and oral cavity cancer (C00-C06) excluding base of the tongue (C01), Females 1982–2008.

Joinpoint analysis for Lip and Oral cavity cancers for men showed an increasing trend of 0.5% per year from 1982–1994 and a larger declining trend by 3.1% per year from 1994 to 2008 (Figure 3). There was a steady increase of 2.3% per year from 1982 to 1996 among females while a sharp decline of 3.0% per year was demonstrated from 1996 to 2008 (Figure 4). When considering both sexes together for the same cancers, an increasing trend with annual change of 0.9% from 1982 to 1995 and declining trend with 2.8% annual change was shown from 1995 to 2008.

When Oropharyngeal cancers (C01, C05.1, C05.2, C09, C10 and C14) are considered separately there was an increase for males from 2.83/100,000 in 1982 to 3.97/100,000 in 2008, for females from 0.79/100,000 in 1982 to 1.01/100,000 in 2008 and for both sexes combined from 1.72/100,000 in 1982 to 2.46/100,000 in 2008.

**Figure 5:**
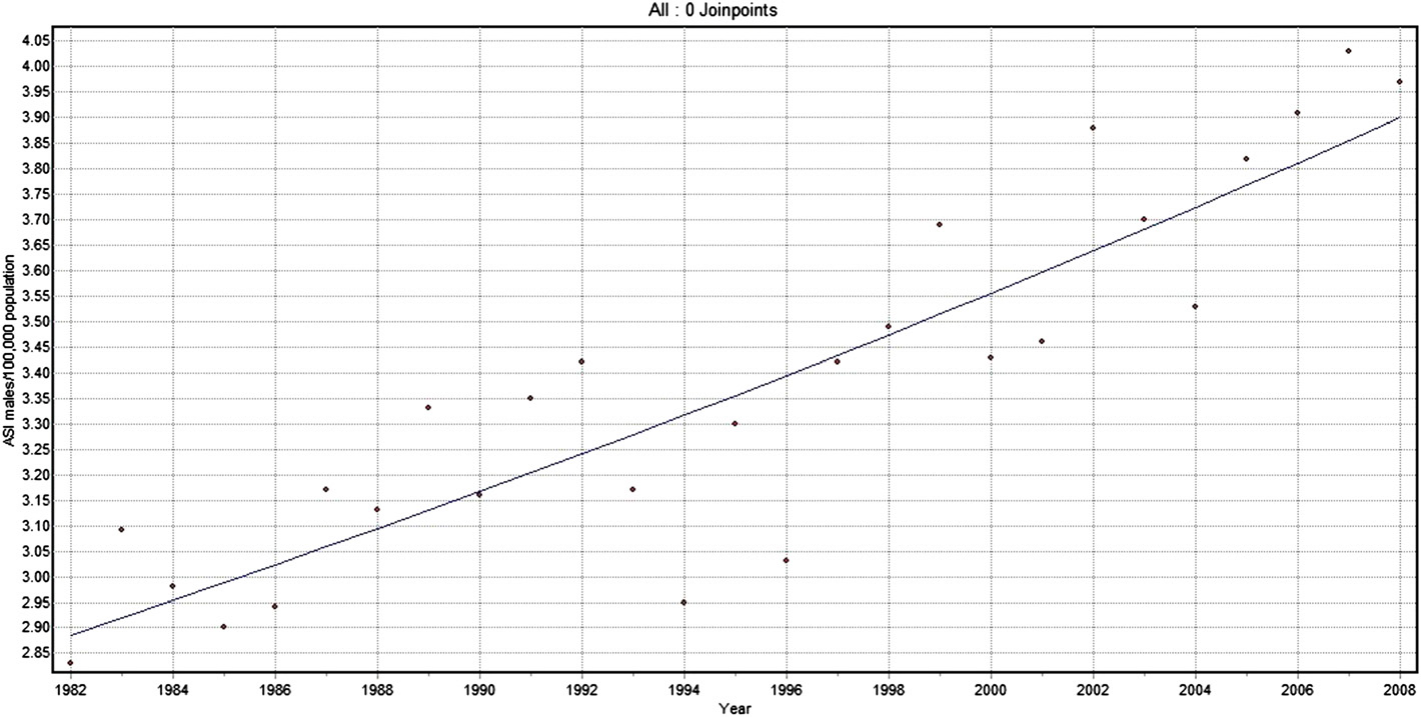
**Trends in incidence of Oropharyngeal cancer (C01, C05.1, C05.2, C09.0, C09.1, C09.8, C09.9, C10 and C14,).** Males 1982–2008.

**Figure 6:**
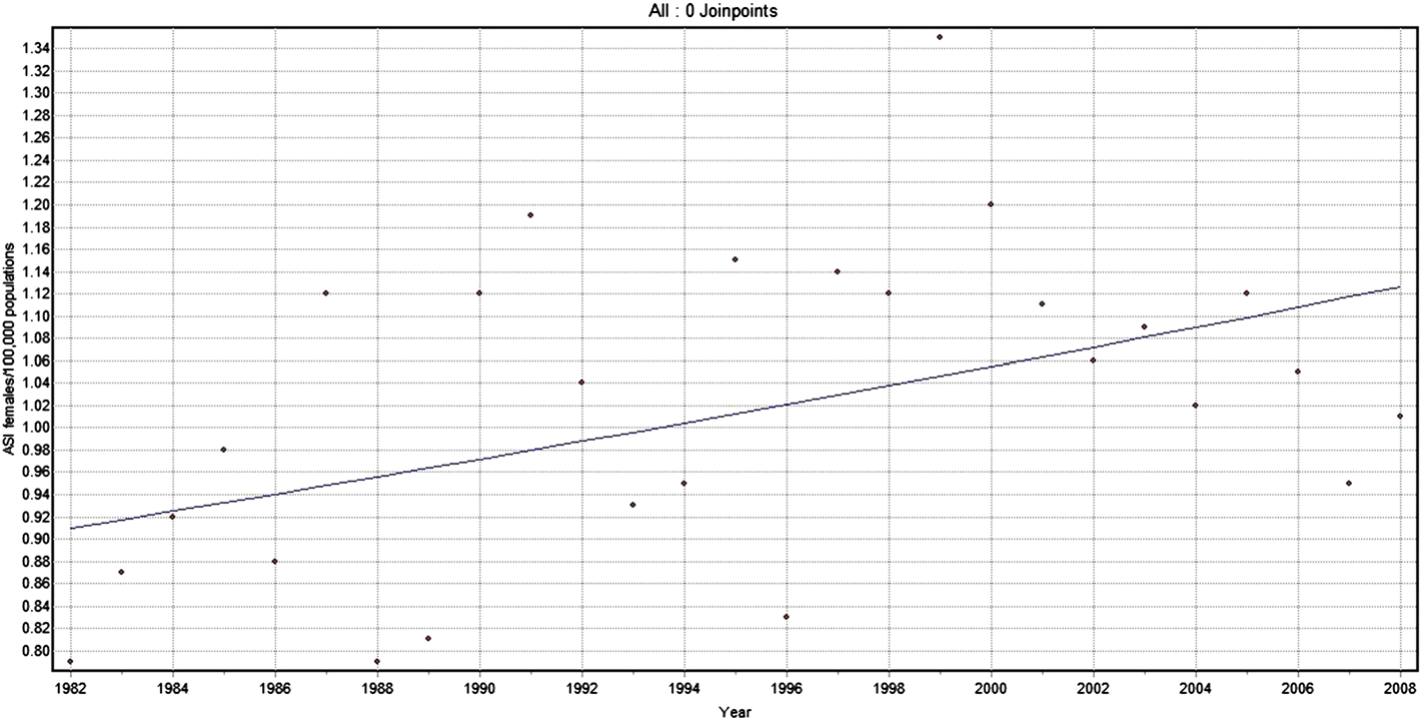
**Trends in the incidence of Oropharyngeal cancer (C01, C05.1, C05.2, C09.0, C09.1, C09.8, C09.9, C10 and C14,). **Females 1982–2008.

Joinpoint analysis for Oropharyngeal cancers for men showed a steadily increasing trend with annual change of 1.2% (Figure 5). There was an increase in trend of 0.8% per year from 1982 to 2008 for females (Figure 6). When both sexes were considered together for the same cancers of the oropharynx, there was a steadily increasing trend at an annual change of 1.2%.

In this study we have analysed the sub-sites of the Lip, Oral Cavity and Oropharynx to identify the main contributors to the overall change in trends of major sites. Table 2 shows the age standardized incidence (ASI) rates by sub-sites: upper lip (ICD10; C00.0 and C00.3); lower lip (C00.1 and C00.4); lip unspecified (C00.2, C00.5,C00.6,C00.8 and C00.9); base of tongue (C01); other and unspecified parts of tongue (C02); gum (C03.0,C303.1 and C03.9); floor of mouth (C04.0,C04.1,C04.8,C04.9); palate (C05.0,C05.1,C05.2,C05.8,C05.9); other unspecified parts of mouth (C06.0,C06.1,C06.2,C06.8, C06.9); tonsil (C09.0,C09.1,C09.8 and C09.9); oropharynx (C10) and ill-defined sites of lip, oral cavity and pharynx (C14) of the cancers for the beginning (1982) and the end (2008) of the study period and the results of the Joinpoint regression analysis for males and females.

**Table 2:** Lips, Oral cavity and oropharyngeal cancer incidence rates for Australia according to sub-sites from 1982–2008 by sex

**Site**	**Sex**	**ASI**		**Joinpoint analysis (1982-2008)**					
		**Year 1982**	**Year 2008**	**Trend 1**		**Trend 2**		**AAPC**	
				**Period**	**APC**	**Period**	**APC**	**1999-2008**	**2004-2008**
**Upper lip ICD10; C00.0 and C00.3**	Male	0.26	0.19	1982-2008	−0.1			−0.1	−0.1
	Female	0.19	0.26	1982-1990	13.1*	1990-2008	−1.3	−1.3	−1.3
**Lower lip ICD10; C00.1 and C00.4**	Male	4.93	3.34	1982-1996	1.5*	1996-2008	−4.2*	−4.2*	−4.2*
	Female	0.68	0.9	1982-1996	6.1*	1996-2008	−4.3*	−4.3*	−4.3*
Lip unspecified** ICD10;C00.2, C00.5,C00.6,C00.8 and C00.9	Male	1.58	0.25	1982-1996	−2.2	1996-2008	−10.5*	−10.5*	−10.5*
	Female	0.13	0.09	1982-1989	12.9*	1989-2008	−6.2*	−6.2*	−6.2*
**Base of tongue ICD10;C01**	Male	0.62	1.03	1982-2008	3.2*			3.2*	3.2*
	Female	0.12	0.22	1982-2003	4.2*	2003-2008	−8.9	−3.3	−8.9
**Other and unspecified parts of tongue ICD10;C02**	Male	1.81	1.61	1982-2008	−1.0*			−1.0*	−1.0*
	Female	0.94	0.79	1982-2008	−0.1*			−0.1*	−0.1*
**Gum ICD10;C03.0,C03.1 and C03.9**	Male	0.22	0.22	1982-2008	0.4			0.4	0.4
	Female	0.21	0.25	1982-2008	1.0*			1.0*	1.0*
**Floor of Mouth ICD10;C04.0,C04.1,C04.8, C04.9**	Male	1.42	0.72	1982-2008	−2.6*			−2.6*	−2.6*
	Female	0.4	0.25	1982-2008	−2.1*			−2.1*	−2.1*
**Palate ICD10;C05.0,C05.1,C05.2, C05.8, C05.9**	Male	0.5	0.4	1982-2008	−1.9*			−1.9*	−1.9*
	Female	0.25	0.29	1982-2008	−0.4			−0.4	−0.4
**Other unspecified parts of mouth ICD10;C06.0,C06.1,C06.2, C06.8, C06.9**	Male	0.39	0.53	1982-2008	0.01			0.0	0.0
	Female	0.34	0.33	1982-2008	0.01			0.0	0.0
**Tonsil ICD10; C09.0,C09.1,C09.8 and C09.9**	Male	1.1	1.9	1982-1995	−0.3	1995-2008	4.3*	4.3*	4.3*
	Female	0.36	0.49	1982-2008	1.1*			1.1*	1.1*
**Oropharynx ICD10; C10**	Male	0.6	0.47	1982-2008	−2.2*			−2.2*	−2.2*
	Female	0.15	0.11	1982-2008	−1.7*			−1.7*	−1.7*
**Ill defined sites of lip, oral cavity and pharynx ICD10;C14**	Male	0.29	0.35	1982-2008	1.5*			−1.5*	−1.5*
	Female	0.09	0.05	1982-2008	−1.0			−1.0	−1.0

Abbreviations

*APC*, Annual Percent Change *AAPC*, Average Annual Percent Change.

*The Annual Percent Change (APC) is significantly different from zero at alpha = 0.05.

*The Average Annual Percent Change (AAPC) is significantly different from zero at alpha = 0.05.

**Include commissure.

Cancer of the lower lip was found to be the dominant anatomical site for both men and women out of all cancers considered in the present study, accounting for 35.3% of all Lip, Oral Cavity and Oropharyngeal cancers. The ASI for males was 4.93/100,000 pa and 3.34/100,000 pa in 1982 and 2008 respectively. This cancer showed an increasing trend from 1982 to 1996 with an annual change of 1.5% and a decline of 4.2% per year thereafter. Although overall ASI was small compared to males, a similar biphasic trend pattern was shown among females, with 6.1% annual increase and 4.3% annual decrease for the same periods.

Cancer of the upper lip was higher among females than males. A slight decline was shown amongst males over the period from 1982 to 2008 with an annual change of 0.1%. In females there was a sharp increase from 1982 to 1990 with an annual change of 13.1% and a decreasing trend thereafter with annual change of 1.3%.

In males, cancers of the base of tongue have shown an increasing trend with annual change of 3.2% from 1982–2008. Although, a similar pattern was shown among females from 1982 to 2003, a decline with 8.9% annual change was shown thereafter. Of the other Oropharyngeal sites considered in this study, tonsillar cancers have shown overall increasing trends for both males and females. Most of the other “sub-sites” have shown overall declining trends.

## Discussion

Our study provides time trends for cancers of individual sub-sites according to the ICD 10 classification for those head and neck neoplasms which have, to a degree, common risk factors. Joinpoint analysis provides a much clearer picture of time trends, in different segments of time, within the overall period concerned. As such we were able to show that significant changes in trends have taken place during this period.

There has been an encouraging decline in lip, oral cavity and pharyngeal cancers overall (ICD10, C00-06, C09, C10 and C14) in Australia in recent decades. However, when cancers of the oropharynx are considered separately, rising trends have been shown, particularly among men, from 1982 to 2008.

We have shown biphasic trends for Lip, Oral Cavity and for Oropharyngeal cancers combined for both men and women. An increasing trend of 0.9% p.a. from 1982 to 1992 and a decline of 1.6% p.a. from 1992 to 2008 were observed in males. Similar biphasic trends were demonstrated among females with a steady increase of 2.0% pa from 1982–1997 and a sharp decline of 2.8% pa thereafter. In a study covering the State of New South Wales, Macfarlane et al., in 1994, reported a similar pattern:. They found increasing incidence of “oral and pharyngeal” cancer from 6.5/100,000 pa to 9.3/100,000 pa among males, and from 2.1/100,000 pa to 3.0/100,000 pa among females, from 1974 to 1986: However, this trend declined thereafter [18]. Another epidemiological study on “lip and oral cavity” (which unfortunately included cancers of the major salivary glands), based on data from the Cancer Registry of Western Australia, reported increasing trends in “oral and pharyngeal cancer” from 1982–1990 at the rate of 14.6/100,000 p.a. for males and 6.2/100,000 p.a. for females and observed declining trends thereafter [21].

Since the second half of the last century reports from many parts of the world on the incidence of “oral cancer” have described declining, stable or increasing rates in different regions or countries [4,9-12,15,26]. Because of the impossibility of linking cause and effect directly, there is no unambiguous explanation for the causes of these trends. However, the most conceivable explanations are life-style changes, particularly changes in smoking rates [27] and spread of HPV infections [10-12].

Overall per capita tobacco consumption in Australia has declined steadily since the latter part of the last century. Among males the estimated prevalence of tobacco use declined from 58% in 1964 to 18% in 2007. In contrast, among females the prevalence of tobacco smoking increased from 28% in 1964 to a peak of 31% in 1980, with a subsequent decline to 15.2% in 2007 [28].

The National Drug Strategy Household Survey 2010 revealed a substantial – almost 40% - reduction in the prevalence of daily smokers in Australia for people aged 14 years or older from 24.3% in 1991 to 15.1% in 2010, [29]. However, increased smoking among females from 1964 to 1980 may have contributed to the statistically significant increase of lip, oral cavity and pharyngeal cancers observed in the present analyses during the 1982–1992 period.

The synergistic effect of alcohol consumption and smoking has been well established [30]. Overall per capita alcohol consumption in Australia in 1960 was estimated at 9.4 L pa. This gradually increased to 13.0 L in 1980 and slowly declined to 10.1 L in 2009 [28]. In 2010, 1 in 5 people in Australia at or over the age of 14 years consumed alcohol at harmful levels [29].

A recent report from France indicated a considerable decrease of upper aero-digestive tract cancers in men, while the same were increased in women over the 25 year period from 1980 to 2005, especially oropharyngeal, palatal and hypopharyngeal cancers: world-standardised incidence rates of lip, oral cavity and pharynx cancers combined declined by 42.9% in men while females showed an increase by 48.6% [31]. Decreasing prevalence of smoking among men in the general population and slightly increasing tobacco smoking in women were suggested as accounting for these changes [31]. Significant declines in the incidence of oral cavity and pharyngeal cancers for both men and women in all races in the USA have been observed over the period from 1977 to 2007, reflecting the steady decline in smoking and alcohol consumption in that nation [32]. Another recent study from the USA reported decreasing trends of oral plus pharyngeal cancers for women with APC of −1.0 from 1992 to 2008. In contrast to women, although men showed a decreasing trend with APC of −1.4 from 1982 to 2006, this turned to a rise of 3% pa from 2006 [15]. Unfortunately it is not possible to separate sub-sites in these data.

Lip cancer has been the dominant site in the oral and oropharyngeal region in Australia, contributing over 36% of cases, of which 90% are cancers of the lower lip. These are more common in males with ASI 4.93/100,000 and 3.34/100,000 in 1982 and 2008 respectively. Although this cancer has shown an increasing trend from 1982 to 1996 with an annual change of 1.5%, a decline of 4.2% per year was observed thereafter. Compared to males the overall ASI was small, but a comparable biphasic trend pattern was observed among females, with 6.1% annual increase and 4.3% annual decrease for the same periods.

Contrary to this Tan (1971), in a countrywide hospital based survey, reported a declining trend in lip cancer (upper and lower lip combined) from 6.5/100,000 in 1959 to 4.9/100,000 in 1964 [17]. He found that cancers of the lower lip were 9.9 times more common among males compared to females, whereas the present study revealed a male to female ratio of only 3:1. However, these are quite old data and, in a hospital-based study, under-reporting is likely.

In a state-based study on lip cancer in Western Australia, Abreu et al. (2009) reported an upward trend with 8.9/100,000 and 2.7/100,000 pa for males and females respectively from 1982 to 2006 [20]. These figures are high compared to the national data reported here, variations in incidence in different states in Australia probably being attributable to differences in the rural/urban population mix and in exposure to risk factors. Lip cancer is much more common in those who live or work outdoors, with direct exposure to sunlight [33,34]. High incidence has long been associated with prolonged exposure to solar radiation, especially in people with fair complexion [33-37]. The lower lip receives considerably more direct sunlight than the upper lip [34]. In contrast, the comparatively low incidence of lower lip cancer among females could be attributed to the protective effect of cosmetics and lower outdoor exposures [38].

The present study revealed that cancer of the upper lip was higher among females than males. Moreover, there was a sharp increase in incidence from 1982 to 1990, an annual change of 13.1%, which started declining thereafter with annual change of 1.3%. In contrast, a slight decline in cancer of the upper lip was observed amongst males over the whole period from 1982 to 2008, with an annual change of 0.1%. As with the present study, significant female predilection for cancers of the upper lip was reported from Western Australia [20]. An almost equal sex distribution of upper lip cancers was reported in another Australia-wide hospital-based study in 1971 [17]. Although no unambiguous explanation for a higher incidence of upper lip cancers among females can be given, differences in exposure factors, particularly increasing prevalence of tobacco smoking among females from the early 1960’s until 1980, may have contributed [28].

In contrast to “lip and oral cavity” cancers, when “oropharyngeal” cancers are considered separately, we have observed increasing trends, throughout the period analysed here. Oropharyngeal cancer has been reported to be increasing significantly and quickly in several countries, particularly in the developed world, and is widely regarded as associated with infection with humanpapillomaviruses of known high oncogenic potential – especially HPV-16 and −18 [10,13,14,39-41]. A recent report from Australia described a significant increase in potentially HPV-associated head and neck cancers in both males and females between 1982 and 2005, with an annual percentage increase of 1.04% and 1.42% for females and males respectively [16]. Our findings are comparable with this, but changes in life-style risk factors, especially smoking and heavy alcohol consumption, and their synergism, will have confounding effects in understanding the causes of cancers in these sites. Varying degrees of exposure may partly explain differences between males and females.

Although overall rates of lip, oral cavity and pharyngeal cancer are currently declining in Australia, these are still high in comparison with many other countries. Efforts to reduce the burden of these cancers remain vital. Further reductions in exposure to lifestyle risk factors: ultraviolet light; all forms of tobacco; excessive alcohol use/abuse; and the consumption of diets rich in antioxidants and minerals, need to be promoted. Sexual hygiene needs to be promoted to reduce the carriage of HPVs in the upper aero-digestive tract: it will be interesting to examine the extent to which current vaccination programs against oncogenic HPVs, at present focused on young women for the prevention of cancer of the uterine cervix, lead to reductions in oropharyngeal cancer in the long term [42,43].

Limitations of our study include the small number of cases in certain sub-sites and subgroups, an inevitability in a nation with a small population (20 million and less during the period under study) spread over a vast geographical area, and that we have not been able to explore differences by ethnic group as we were not permitted access to this information for ethical reasons.

## Conclusions

This study has demonstrated encouraging reductions in the combined incidence of Lip, Oral cavity and Oropharyngeal Cancers over the study period. This is consistent with the steady decline in known life style risk factors such as tobacco smoking and alcohol drinking. Although this is overall good news, work is needed to reduce the incidence further, since the rates are still high compared to several other countries. While some cancers have shown a particularly steep decline, notably cancers of the lower lip, there has been a disturbing increase of cancers of the tonsil and base of tongue, consistent with global trends, and likely to be related to increasing HPV infections of the oropharynx. Revised public health messages and continued surveillance is required to negate these rising trends. Moreover, it is imperative to undertake studies to identify particularly vulnerable groups in Australian society.

## Competing interest

The authors declare that they have no competing interests.

## Authors’ contributions

NWJ initiated the study and negotiated access to the national databases. AA created the working files and performed the Joinpoint analyses. Both authors shared equally in interpretation of the data and in manuscript preparation. Both authors read and approved the final manuscript.

## Pre-publication history

The pre-publication history for this paper can be accessed here:

http://www.biomedcentral.com/1471-2407/13/333/prepub
